# Short-term impact of oral hygiene training package to Anganwadi workers on improving oral hygiene of preschool children in North Indian City

**DOI:** 10.1186/1472-6831-13-67

**Published:** 2013-11-27

**Authors:** Sonika Raj, Sonu Goel, Vijay Lakshmi Sharma, Naveen Krishan Goel

**Affiliations:** 1 Centre for Public Health, Panjab University, Sector 14, Chandigarh 160014, India; 2 School of Public Health, Post Graduate Institute of Medical Education and Research, Sector 12, Chandigarh 160012, India; 3 Department of Community Medicine, Government Medical College and Hospital, Sector 32, Chandigarh 160035, India

**Keywords:** Snyder’s test, Oral health training, Anganwadis, Caries activity

## Abstract

**Background:**

Globally, dental caries is categorized in the list of public health problems in preschool children. In India, lack of availability and affordability of oral health enhances the cost of treatment and care. Empowering community workers like anganwadi workers (AWWs) in oral health, and providing basic oral health awareness to the mothers through them can be feasible model. So, the present study was conducted to evaluate the short-term impact of Oral Hygiene Training Package (OHTP) to AWWs on improving oral hygiene of preschool children.

**Methods:**

This before and after comparison field trial was done in Anganwadi centres (AWCs) of Chandigarh city, India. 534 children aged 36-72 months attending 21 AWCs were examined before and after imparting trainings to AWWs. OHTP was administered to AWWs, which consisted of power-point presentation and demonstrated the skills like proper brushing technique, plaque disclosure, flossing technique, gum massaging etc. The AWWs later imparted training to mothers in their respective AWCs. Post intervention data was collected after three months.

Outcome measures were improvement in oral health status (plaque, debris, gingival health), oral habits (brushing, rinsing) and decrease in caries activity (Snyder test).

**Results:**

Prevalence of dental caries was found to be 48.3%. Only 4.1% of the population reported brushing twice which increased significantly to 9.9% post-intervention (p = 0.000). There was a significant decrease in debris (78.3% to 54.1%), and stage-1 plaque (75.5 to 66.5%) in the oral cavity. Caries activity by Snyder’s test decreased from 48.2% to 31.2% (p = 0.01) post-intervention.

**Conclusions:**

Controlled trials of using AWWs to improve oral hygiene appear to be justified.

**Trial registration:**

CTRI/2012/07/002786

## Background

Globally, dental caries is categorized in the list of public health problems in the children aged 3-6 years [1]. The lack of availability and affordability of oral health services especially in developing country like India not only results in aggravation of the disease but also enhances the cost of treatment and care. It has been observed across various countries that basic health care workers and parents have limited knowledge about causes and prevention of the most common oral diseases [2-5]. Control of oral diseases is only possible if services are oriented towards primary health care and prevention.

*Anganwadi* workers (AWW), the grass root workers (serves around 1000 population per AWW) have successfully demonstrated their useful role in developing healthy habits in early childhood viz. correct brushing techniques and hand washing through non-formal education methods (learning by play way method) [6]. At the *anganwadi*s, monthly meeting of mothers serve a platforms for disseminating health education to mothers regarding immunization, breast feeding, institutional delivery, postnatal care etc. Empowering community workers like AWW in oral health, and providing basic oral health awareness to the mothers through them can be feasible model for a developing country like India; where oral health is not a priority in the primary health care as yet.

The present study was conducted with the objective to evaluate the short-term impact of Oral Hygiene Training Package (OHTP) to *Anganwadi* workers on improving oral hygiene of preschool children.

## Methods

### Study area

The study was conducted in Chandigarh city, which is a capital of two states and also a Union Territory. It lies in northern part of India and is spread in a geographical area of 114 sq kms. The city boosts of health indicators, which are amongst the best in India [7]. There are a total of 423 Anganwadi Centres (AWCs) in Chandigarh which are administratively divided into 3 projects catering to around 35,000 preschool children [8].

### Study design and sampling

This short term interventional study was carried out for a period of 7 months. Baseline data collection was done in month of September-October, 2010 followed by training to AWW in month of November, 2010. Endline data (Post-intervention) was collected after three months (February, 2011). The intervention duration of 3 months between pre and post test was chosen to observe the short term impact of OHTP on oral hygiene of preschool children.

The sample size for the study was determined by EPI-INFO WHO package. Taking alpha error to be 0.05 (likelihood of association by chance alone as less that 5%), beta error 0.20 (i.e. statistical power of the study as 80%), assuming baseline poor oro-dental hygiene of the children as 40% and expected improvement in oro-dental hygiene by oral health training package to be approximately 15% (effect size), drop-out rate as 10%, a sample size of 495 students was estimated. Assuming that an anganwadi will contain around 20-25 children in age group of 36-72 months, a total of 21 anganwadis (Seven AWCs from each project) were selected by simple random sampling using the table of random numbers.

### Study population

**Figure 1:**
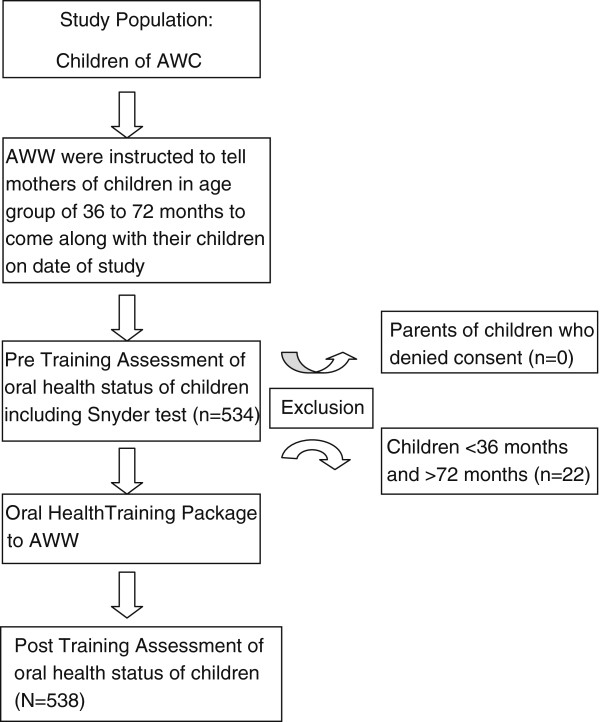
Flowchart depicting study design and selection process of study population.

All the children aged 36-72 months attending the selected *Anganwadi* Centre (AWCs) on the date of visit were included in the study. The consent of parents was taken prior to examination of their wards. The children less than 36 months or over 72 months of age or whose parents denied consent were excluded. All the children in selected *anganwadis* were examined for dental morbidity (i.e. dmft, gingival bleeding and plaque index) using pretested semi structured proforma before and after imparting trainings to AWW (Figure 1).

### Intervention (oral hygiene training package)

The AWWs were provided with Oral Hygiene Training Package (OHTP) by the principal author of the study. Repeated examinations were performed on two hundred and fifty children to confirm intra-examiner reliability. The kappa value was observed to be 0.72. In the package, they were imparted knowledge by means of a power-point presentation, posters, photo albums, dentoform and plaster models, which focused on oral health care and hygiene practices; functions of teeth; dentitions and their significance; dietary habits and their effects on oral hygiene; importance of a tooth friendly diet; etiology and prevention of dental caries; injurious oral habits; influence of oral health on general health; importance of twice daily brushing; use of fluoride toothpaste; common dental/ gum disorders; bottle feeding and its effect on nursing bottle caries; intelligent use of sweetened things; tongue cleaning; mouth rinsing; proper tooth-brushing technique and importance of a regular dental visit. The flossing was however discouraged in the age-group of 36-72 months. In addition, they were provided an on-the-spot practical demonstration of tooth brushing and flossing methods using dentoform models. In order to have a better understanding about plaque, disclosure of dental plaque was also conducted. The subjects were later told to demonstrate the tooth brushing and flossing on the study models, which was monitored by the investigator. All AWWs were distributed a self designed poster and story on oral hygiene to be recited to children in their AWCs every day. All the training material was translated in local language for better understanding of AWWs. The OHTP was imparted to AWWs in eight batches having 50 AWWs per batch with an average duration of around two hours per batch. The training continued for four days. The training module, poster and story were developed by main researcher in collaboration with the professionals in field of dental public health.

The AWWs were later distributed a schedule of training, wherein, they imparted training to mothers in their respective AWCs on weekly basis. A total of 12 meetings took place at each AWCs, which were monitored by a team from Department of Health and Family Welfare, U.T, Chandigarh along with the researchers of the study. Prior to conduction of meeting, the mothers were informed to participate. During meeting, they were briefed about OHTP by means of training module, posters and story distributed to them.

A pretest and post-test of knowledge (by questionnaire) and skills (by observational checklist) was done before and after administration of OHTP. The reliability of study tools was checked by ‘test-retest’ concept i.e. the tools were pretested in population different from study area and checked for consistent results. The tools were validated for linguistic (questionnaire asked in similar language i.e. local language) and content validity.

### Clinical examination

All the children in study group were clinically examined by the qualified dentist (principal author) accompanied by a trained dental assistant who recorded the data on a standardized form. The training and calibration of the examining qualified dentist was carried out at Oral Health Sciences Department of a tertiary care government hospital. The examination was carried out under natural sunlight using a WHO referenced disposable plane mirror, dental explorer, periodontal probe [9]. The child was made to sit on a bench and the examiner at its head. Caries was recorded based on dmft (decayed, missing, filled teeth) index using codes and criteria as described by WHO [10].

Presence of plaque was observed on upper anterior teeth without the use of disclosing agent. The explorer was passed over the labial surface of anterior teeth; if plaque was present on gingival one third then considered as stage 1, if extended to middle one third then stage 2 and if extending to whole tooth area then it was taken as stage 3. The Debris Index was used to measure debris in children as an objective measure of brushing behavior. If debris was detected on two or more teeth on both lingual and buccal surfaces or the score ranges from 1.8-3, the children is said to be positive for debris [11]. The presence/absence of gingival bleeding was assessed by positioning the periodontal probe in the gingival crevice at a depth of 1 mm and then passing it around the gingival collar of the tooth. The tooth was observed for 15 to 30 seconds following probing and was scored as being positive for gingival bleeding [9]. The presence of calculus was also determined visually on buccal and/or lingual surfaces of the teeth.

The mothers were also interviewed regarding oral habits, frequency of toothbrushing, rinsing of mouth, medium for cleaning teeth of their children before and after the training. The socioeconomic status evaluation of family of respondent was done on the basis on the revised Kuppuswamy Socioeconomic Status Scale [12].

Snyder test, a calorimetric caries activity test, was also done among the children. Samples of saliva (0.2 ml) of children were taken in tubes with Snyder test agar media and incubated at 37 degree C. The readings were taken at 24, 48, 72 & 96 hrs. Colour changes were compared with an un-inoculated control tube for three days which is interpreted as: Positive colour change in 24 hours: marked caries activity, positive colour change in 48 hours: moderate caries activity, negative colour change in 48 hours: slight caries activity and negative colour change in 72 hours: negative caries activity [13].

### Ethical considerations

Ethical approval to conduct the study was obtained from the Institutional Ethical Committee of Post Graduate Institute of Medical Education and Research, Chandigarh, prior to the implementation of the study. The consent of the Project Officer, Integrated Child Development Services (ICDS) (In-charge of AWCs); AWWs and parents of the children were also obtained before commencing the study.

### Evaluation of intervention

The outcome indicators of the study includes improvements in oral hygiene practices (frequency of tooth brushing, mouth rinsing after meals) and also changes in plaque index scores, gingival index scores, debris index and caries activity in the intervention group before and after training to AWW.

### Data management and processing

The data were entered in Microsoft Excel 2007 and statistically analysed using SPSS version 16. Descriptive statistical analysis was carried out using Chi-square test, Paired t-test and ANOVA. The comparison (mean and percentage changes) between the pre and post intervention score was done for oral hygiene practice, debris index, plaque index scores and gingival index scores. The impact of the OHTP was also evaluated on the basis of change in Snyder test results post-intervention as compared to pre-intervention. Significance was assessed at 5% level of significance.

## Results

A total of 534 & 538 children respectively were examined before and after training of AWWs. No significant difference in the demographic characteristics of study population between pre-intervention and post-intervention was found (Table 1). The average age of respondent children before and after intervention was 44 months.

**Table 1:** Demographic profile of the study population before and after training package

**Socio-demographic variables**	**Pre training**		**Post training**	
	**(n = 534)**		**(n = 538)**	
	**N**	**(%)**	**N**	**(%)**
**Age group (in months)**				
36-48	259	(48.5)	274	(50.9)
49-60	188	(35.2)	177	(32.9)
61-72	87	(16.3)	87	(16.2)
**Gender**				
Female	263	(49.3)	246	(45.7)
Male	271	(50.7)	292	(54.3)
**Set up**				
Urban	258	(48.3)	247	(45.9)
Peri-urban	124	(23.2)	120	(22.3)
Rural	152	(28.5)	171	(31.8)
**Socio-economic status**				
Upper	1	(0.2)	0	(0.0)
Upper middle	17	(3.2)	20	(3.7)
Lower middle	87	(16.3)	114	(21.2)
Upper lower	415	(77.7)	402	(74.7)
Lower	14	(2.6)	2	(0.4)

During pre-intervention, prevalence of dental caries was found to be 48.3% (n = 258). The mean dmft score of study population was slightly higher in males (2.14) as compared to females (2.08) but the difference was found to be statistically insignificant (p > 0.05). There were no cases of filled teeth in any of the children examined. The mean dmft was found to be significantly (p < 0.05) higher in 49-60 months age group (2.72) as compared to 61-72 months (2.53) and 36-48 months age group (1.53). Dmft per affected child was highest (4.74) in age group of 49-60 months followed by 36-48 months age group (3.99). The dmft score was highest (2.25) in urban settings and in lower socio-economic strata (3.50) but the difference was statistically insignificant (p > 0.05) (Table 2).

**Table 2:** Mean dmft scores of the study population

**Socio-demographic variables**	**dmft mean ± SD**	**Children with decayed teeth**		**p value**
		**N**	**%**	
**Age group (months)**				
36-48	1.53 ± 2.77	97	37.4	0.000^*^
49-60	2.72 ± 3.67	106	56.3	
61-72	2.53 ± 3.00	55	63.2	
**Gender**				
Female	2.08 ± 3.23	118	44.8	0.806
Male	2.14 ± 3.17	139	51.2	
**Setup**				
Urban	2.25 ± 3.51	124	48.1	0.438
Peri urban	2.15 ± 2.99	67	54.1	
Rural	1.84 ± 2.77	73	48	
**Socio-economic status**				
Upper	-	0	0	0.642
Upper Middle	1.94	7	41.1	
Lower Middle	1.85	39	44.8	
Upper Lower	2.13	202	48.6	
Lower	3.50	10	71.4	

*Significant at 0.05 level.

Only 4.1% of the population reported to brushing twice or more daily before the intervention, which increased significantly to 9.9% post-intervention. Before the intervention, around 14% children never brushed their teeth in their lifetime which reduced to half (7%) after the intervention. The rinsing of mouth post-meals also increased significantly from 39.5% to 52.2% (Table 3).

**Table 3:** Self reported oral hygiene practices among children examined before and after training

**Oral hygiene practices**	**Pre-training**		**Post-training**		**p-value**
	**(n = 534)**		**(n = 538)**		
	**N**	**(%)**	**N**	**(%)**	
**Brushing (per day)**					
Once	252	(47.2)	251	(46.7)	0.908
Twice or more	22	(4.1)	53	(9.9)	0.000^*^
Sometimes	186	(34.8)	195	(36.2)	0.674
Never	74	(13.9)	39	(7.2)	0.000^*^
**Medium of cleaning**					
Tooth brush	455	(85.2)	485	(90.2)	0.017^*^
Tooth powder	5	(0.9)	14	(2.6)	0.066
No	74	(13.9)	39	(7.2)	0.000^*^
**Rinse mouth (per day)**					
Yes	211	(39.5)	281	(52.2)	0.000^*^
No	282	(52.8)	251	(46.7)	0.050^*^
Sometimes	41	(7.7)	6	(1.1)	0.000^*^

*Significant at 0.05 level.

There was a significant decrease in debris (78.3% to 54.1%), and stage-1 plaque (75.5 to 66.5%) in the oral cavity in post-intervention as compared to pre-intervention. However, gingival health status, and thumb sucking showed no significant improvement (Table 4).

**Table 4:** **Oral health status of children in *anganwadi *** **centres before and after training of Anganwadi workers**

**Oral diseases/habit**	**Pre-training**		**Post-training**		**p-value**
	**(n = 534)**		**(n = 538)**		
	**N**	**(%)**	**N**	**(%)**	
**Debris**	418	(78.3)	291	54.1	0.000^*^
**Flourosis**	30	(5.6)	37	(6.9)	0.468
**Plaque**					
Stage 1	403	(75.5)	358	(66.5)	0.001*
Stage 2	113	(21.2)	166	(30.9)	0.000*
Stage 3	4	(0.7)	14	(2.6)	0.033*
**Oral habit (thumb sucking)**	46	(8.6)	40	(7.4)	0.549
**Gingival health status**					
Healthy	519	(97.2)	509	(94.6)	0.048^*^
Bleeding	9	(1.7)	12	(2.2)	0.671
**Calculus**	6	(1.1)	17	(3.2)	0.036^*^
**Mean dmft**	2.1 ± 3.20		1.9 ± 1.4		0.060
**Prevalence of caries**	48.3%		47.8%		0.071

*Significant at 0.05 level.

Caries activity amongst children was determined by Snyder test. It was observed that caries activity among children significantly decreased from 48.2% (n = 241) pre-intervention to 31.2% (n = 168) post-intervention. The prevalence of children with very high caries activity (Snyder test positive in 24 hours) also decreased from 19.8% to 14.9% post-intervention (Table 5).

**Table 5:** Snyder test at different time intervals before and after training of AWW

**Snyder test**	**Pre-training(n = 499)**		**Post training (n = 538)**		
	**N**	**%**	**N**	**%**	**p-value**
**Positive after 24 hours**	99	19.8	80	14.9	0.126
**Positive after 48 hours**	84	16.8	57	10.5	0.016^*^
**Positive after 72 hours**	58	11.6	31	5.7	0.003^*^
**Negative after 72 hours**	258	51.7	370	68.7	0.000^*^

*Significant at 0.05 level.

## Discussion

The present study focuses on improving oral hygiene of preschool children by providing oral health education to *anganwadi* workers, who further disseminate the knowledge to mothers and children attending AWCs. Training the AWWs on oral health will ensure that a wider section of society can be educated on basic oral hygiene with minimum utilization of resources. Moreover, such training will ensure that the health promoting behaviors learnt in early childhood are deeply ingrained in the society and are resistant to change. In the light of the scarce resources in dental public health and the burden of oral diseases in India, a prevention-oriented oral healthcare policy would be more advantageous than the curative approach [14].

Key findings of this study were short-term reductions in debris, stage-1 plaque and caries activity besides improvements in self-reported oral hygiene practices. The lecture-demonstration method used in the present study for imparting oral health education to AWW was similar to other studies [15,16]. Studies have also demonstrated that regardless of age, children submitted to a preventive oral health programme have better oral hygiene status as compared to those who are not enrolled in any such programme [15-18]. The similar package used in a study by Thomas et al. showed significant improvement in various oral habits and hygiene of children [19]. A study by Haleem et al. has concluded that various strategies (dentist-led, teacher-led and peer-led strategies) of oral health education are equally effective in improving the oral health knowledge and oral hygiene status of adolescents [20]. Similar results were observed in other studies [16,17,21-23]. Albandar et al. [24] have reported a long-term impact of oral health education programme on reduction of plaque and gingival inflammation in adolescents. Their programme included regular follow-ups and constant communication with parents. However, the present study could not evaluate the longer term impact due to its shorter duration.

However, many studies have shown that educational interventions are not effective in behavior change. A systematic review by Kay et al. (1998) has concluded that the various oral health promotion measures have not shown to be effective in altering the behavior and clinic indices of disease. The oral health promotion which brings about the use of fluoride is however effective for reducing caries [25]. Watt et al. (2005) reviewed extensive collection of public health policy documents produced by WHO, and demonstrated that public health strategies should tackle the underlying social determinants of oral health through the adoption of a common risk approach rather than isolated interventions which merely focus on changing oral health behaviors. It also concluded that most significant limitation of largely educational interventions is that they fail to achieve sustainable improvement in oral health [26]. The studies which have either used water fluoridation or topical fluorides (in toothpastes or mouth rinses or gels) have demonstrated specific reduction in incidence of caries [27,28].Overall, the apparent effectiveness of health education to improve oral health appears limited in better designed studies.

In the present study, the tooth brushing frequency was poor in the study subjects, which improved (4.1% to 9.9%) after the intervention. Similar findings were observed in other studies [2,15,16,29,30]. Tewari et al. [31] concluded that practice of brushing once and twice daily increased in children after imparting a training package about oral health measures to the anganwadi workers and school teachers. Other studies have documented an improvement in dental hygiene status of children irrespective of type of manpower (viz AWW, school teachers, dental professionals etc.) involved in training of parents [17,18].

A study conducted among children, aged between 4-6 years in turkey by Tanboga et al. used modified Snyder test to detect caries activity and found that around 46% of the children had positive results at the end of the 48 hours, which indicated a mild caries activity [32]. Similar results were found in present study wherein, moderate caries activity was found in 16.8% of children. The results suggest that Snyder test can be used as a part of routine assessment of dental health of pre-school children to diagnose children with high caries activity for prioritization of their dental care and management.

## Conclusions

Empowering basic level healthcare workers through existing primary healthcare infrastructure and outreach mechanisms may provide an effective, replicable mechanism of providing primary preventive oral healthcare to the community. However, for knowledge to be translated to positive practice and sustained behavior change, concerted efforts and long term orientation trainings, follow-up and evaluation is necessary. A note of caution must be exercised in generalizing the results of study. The study is uncontrolled and so any improvements in health may be due to the children and parents responding to the assessments. Similarly, a lack of controls and blinding means that there may be measurement bias. Further, as pointed out by the authors, the information collected about the oral hygiene practices in the study was based on self reporting by mother, which might lead to information bias (over reporting) considering tooth brushing as a socially desirable behavior. Long term effects on oral health and sustenance in behavior change could not be ascertained due to short duration of study. A large community trial is recommended to substantiate the findings of study and ascertain the feasibility of educational intervention in improving the oral hygiene of children.

## Competing interests

The authors declare that they have no competing interests.

## Authors’ contributions

SR formulated the study design, participated in data acquisition, analysis and drafted the whole manuscript. SG conceived the study, edited the manuscript and assisted in the analysis of the study. VLS contributed in the definition of intellectual content, interpretation of the results and edited the manuscript. NKG supervised the data analyses and interpretation, edited and gave the final approval of the manuscript. All authors read and approved the final manuscript.

## Pre-publication history

The pre-publication history for this paper can be accessed here:

http://www.biomedcentral.com/1472-6831/13/67/prepub
